# Development of a predictive model for major adverse cardiac events in a coronary artery bypass and valve population

**DOI:** 10.1186/1749-8090-8-177

**Published:** 2013-07-30

**Authors:** Christine R Herman, Karen J Buth, Jean-François Légaré, Adrian R Levy, Roger Baskett

**Affiliations:** 1 Division of Cardiac Surgery, Queen Elizabeth II Health Science Center, Halifax, NS, Canada; 2 Department of Community Health and Epidemiology, Dalhousie University, Halifax, NS, Canada

**Keywords:** Cardiac surgery, Predictive model, Outcomes

## Abstract

**Background:**

Quality improvement initiatives in cardiac surgery largely rely on risk prediction models. Most often, these models include isolated populations and describe isolated end-points. However, with the changing clinical profile of the cardiac surgical patients, mixed populations models are required to accurately represent the majority of the surgical population. Also, composite model end-points of morbidity and mortality, better reflect outcomes experienced by patients.

**Methods:**

The model development cohort included 4,270 patients who underwent aortic or mitral valve replacement, or mitral valve repair with/without coronary artery bypass grafting, or isolated coronary artery bypass grafting. A composite end-point of infection, stroke, acute renal failure, or death was evaluated. Age, sex, surgical priority, and procedure were forced, a priori, into the model and then stepwise selection of candidate variables was utilized. Model performance was evaluated by concordance statistic, Hosmer-Lemeshow Goodness of Fit, and calibration plots. Bootstrap technique was employed to validate the model.

**Results:**

The model included 16 variables. Several variables were significant such as, emergent surgical priority (OR 4.3; 95% CI 2.9-7.4), CABG + Valve procedure (OR 2.3; 95% CI 1.8-3.0), and frailty (OR 1.7; 95% CI 1.2-2.5), among others. The concordance statistic for the major adverse cardiac events model in a mixed population was 0.764 (95% CL; 0.75-0.79) and had excellent calibration.

**Conclusions:**

Development of predictive models with composite end-points and mixed procedure population can yield robust statistical and clinical validity. As they more accurately reflect current cardiac surgical profile, models such as this, are an essential tool in quality improvement efforts.

## Background

Quality improvement initiatives (QI), a cornerstone of cardiac surgery, have largely relied on predictive models to advance the quality of care cardiac surgical patients receive. For the last two decades, coronary artery bypass grafting (CABG) has dominated clinical practice in cardiac surgery, and therefore the majority of quality improvement initiatives have focused on surgical outcomes following isolated CABG surgery [1-4]. More recently, however, there has been an increase in valve and valve-CABG cases in cardiac surgery [5] and existing predictive models for isolated CABG may not accurately reflect current practice profiles. These models may achieve great statistical validity but lack clinical validity as the models are applicable to only a decreasing percentage of the clinical practice. In order to maintain continued success in quality improvement, it is important to delineate risk profiles for a group of mixed procedures, including CABG, valve, and valve + CABG, that characterize current clinical practice.

To accommodate the shift in the profile of cardiac surgical practice, isolated valve and valve plus CABG models have been developed [5-10]. Some debate exists regarding the validity of developing models with hetero-geneous procedures with some advocating for single procedure models [7,9,10]. Homogeneity within the population allows for simplicity of model development with improved reliability but limits sample size available for development and validation [5]. Although mixed procedural models may be confounded or biased by different pathophysiological and risk profiles, they improve sample size [5] and increase their relevance to current surgical practice.

Largely, mixed procedural models have focused on aortic/mitral or valve +/− CABG, with few CABG, valve and CABG +valve models present in the literature. The EuroSCORE model, one of the mostly widely used cardiac surgical prognostic models, has successfully achieved both statistical and clinical relevance as it applies to a mixed surgical practice including CABG, valve, and CABG + valve. However its development and validation cohort were derived from a population sample that is over fifteen years old [11] limiting its applicability to a contemporary surgical practice. Recently, the EuroSCORE II updated its risk model obviating some of the limitations of the original model [12]. However, the performance of this heterogeneous procedure model performed poorly in risk prediction for several of its component procedures types [13,14]. In addition, both the EuroSCORE and the EuroSCORE II are only a mortality model.

Many models describe death as a lone end point, despite that fact that cardiac patients experience a variety of relevant morbidity. These alternate surgical outcomes are also important quality indicators for cardiac surgical care [15] and targets for QI initiatives. Subsequently, modeling composite end points such as, major adverse cardiac events (MACE), that included both important morbidity and mortality may provide more insight to the surgical outcomes experienced by patients as well as increase statistical power in low frequency end points.

Comprehensive models that include most major cardiac surgical procedures may allow for better understanding of patient risk profiles and facilitate quality improvement initiatives directed towards the majority of patients seen in current practice. The objective of this paper is to develop a CABG, valve, CABG + valve morbidity and mortality (MACE) model that can be applied to the majority of patients undergoing cardiac surgery.

## Methods

### Data source and study population

This study is a retrospective cohort design. The Maritime Heart Center Cardiac Surgery Registry is a detailed clinical database housed at the Queen Elizabeth II Health Science Center (QEII HSC) Halifax, Nova Scotia, an academic tertiary care centre performing 1200 cardiac cases per year. It includes pre-, intra-, and post-operative data prospectively collected on all cardiac surgical cases performed at the QEII HSC from 1995 to present. Trained abstractors collect data, and a database administrator maintains the registry. The database is audited annually.

The model development cohort included all patients undergoing isolated coronary artery bypass grafting (CABG), isolated aortic valve replacement, isolated mitral valve repair or replacement with or without concomitant CABG performed at the QEII HSC since 2004 to 2009. The development cohort was restricted to these years in order to maintain a modern population relevant to current clinical practice.

The primary outcome was a composite end point defined as Major Adverse Cardiac Events (MACE) that included in-hospital death, stroke (persisting at discharge or transient), acute renal failure (new post-operative renal failure or acute on chronic (>50% increase from baseline creatinine)), or infection (sepsis, pneumonia, or deep sternal wound infection).

### Statistical methods

#### Variable selection

Candidate variables for model development included the following preoperative characteristics: age, sex, diabetes, frailty, chronic obstructive pulmonary disease, redo sternotomy, atrial fibrillation, hemoglobin, peripheral vascular disease, cerebro-vascular disease, creatinine, ejection fraction < 40%, New York Heart Association classification (NYHA I-IV) and body mass index (BMI). Surgery-related data such as urgency of surgery, and procedure type were also included. These candidate variables were chosen a priori and selected through rigorous review of the literature [1-9,16-18] (Table 1).

**Table 1:** Model variable definitions

**Variable**	**Definition**
Age	Patient age at the time of surgery
Gender	Male or Female
Body mass index	Calculated in kilograms and centimeters.
Diabetes	Any history of Diabetes Mellitus, regardless of duration
Pre-op Afib	Any previously documented history of Atrial Fibrillation
COPD	Any preivous documented history of Chronic Obstructive Pulmonary Disease
CVD	Any Transient Ischemic Attack, Cerebrovascular Accident/Stroke, history of cerebrovascular surgery, or any carotid disease.
PVD	Whether the patient has Peripheral Vascular Disease, as indicated by claudication; amputation for arterial insufficiency; aorto-iliac occlusive disease reconstruction; peripheral vascular bypass surgery, angioplasty, or stent; documented AAA.
Frailty	Any deficiency in the Katz index of Activities of Daily Living (independence in feeding, bathing, dressing, transferring, toileting, and urinary continence), as well as independence in ambulation (no walking aid or assist required) or any clear evidence of a previous diagnosis of dementia by a physician.
EF<40	Ejection fraction measured less than 40% by any modality.
NYHA (I-IV)	New York Heart Association Class. I = Patients with cardiac disease but without limitation of physical activity. II = Patients with cardiac disease resulting in slight limitation of physical activity (fatigue, palpitations, dyspnea, or anginal pain). III = Patients with cardiac disease resulting in marked limitation of physical activity. IV = Patients with cardiac disease resulting in inability to carry on any physical activity without discomfort. Symptoms of cardiac insufficiency or of the anginal syndrome may be present even at rest.
Hemoglobin	Most recent hemoglobin level prior to day of surgery.
Pre-op creatinine	Highest preop serum creatinine for this admission.
Redo sternotomy	Any history of previous surgery that traversed the anterior mediastinum.
Surigical priority	Elective [stable at home], in-house [requiring hospitalization until the time of surgery], urgent [requiring surgery within 24 hours to minimize further clinical deterioration], or emergent [no delay in surgery].
Procedure	Any Coronary artery bypass grafting, aortic valve replacement or repair with/without CABG, or Mitral valve replacement or repair with/without CABG.

Multi-colinearity of candidate variables was assessed with variance inflation factor (VIF) (Additional file 1: see Statistical Methods). The linear relationships of the natural variables and their transformations were assessed through locally weighted scatterplot smoothing (LOESS) regression [19] (see Additional file 1: Linearity and Transformations and Figure 2-3). The WHO classification of BMI was used [20].

#### Model evaluation

A multivariate logistic regression analysis was used to describe MACE.

Age, sex, procedure type and surgical priority were chosen a priori and forced into the model. Stepwise selection was implemented for the remainder of the candidate variables. The concordance statistic and −2 log likelihood were evaluated to assess the contribution of each variable to the model. If a variable did not contribute to an increase in the C or −2 log likelihood statistic it was not retained in the model.

Model discrimination was determined using the concordance statistic [21]. Model calibration was assessed by the Hosmer-Lemeshow goodness-of-fit statistic [22,23] as well as calibration plots [24]. Deciles of observed and predicted probabilities of MACE were plotted for the calibration plots [5]. Bootstrap procedure was used to internally validate the model.

All statistical analysis was performed using SAS software version 9.2 (SAS, Cary, NC).

Approval for conducting this study was obtained from the Institutional Review Board of the Capital District Health Authority. The requirement to obtain informed consent was waived under Section 2.1c of the Tri-Council Policy Statement. All personal identifiers were stripped prior to data analysis to ensure patient anonymity and confidentiality.

The authors had full access to the data and take full responsibility for its integrity. All authors have read and agree to the manuscript as written.

## Results

### Population

A total of 4,270 patients underwent CABG, valve (aortic valve replacement, mitral valve replacement, or mitral valve repair) or CABG + valve at the QEII HSC from Jan 2004 to Dec 2009. The model cohort was 65% of the total case volume (6,525) performed during the study period. The distributions of risk factors in the development cohort are displayed in Table 2. The prevalence of MACE in this cohort was 15.7% (n=669). The prevalence of MACE was higher in the CABG plus valve group (32%, n=155) than in the isolated procedures (CABG 13%, n=416; Valve 14%, n=98). The frequencies of MACE for each procedure as well as the components of MACE are summarized in Table 3.

**Table 2:** Distribution of risk factors in the model development cohort

**Variable**		**All Procedures**	**CABG**	**AVR/MVR/MVrpr**	**CABG + Valve**
		**n=4270**	**n=3095**	**n=696**	**n=479**
		**(%)**	**(%)**	**(%)**	**(%)**
Age		67 (IQR 59–74)	66 (IQR 58–73)	67 (IQR 57–75)	74 (IQR 67–80)
Female		25	21	39	31
BMI (kg/m^2^)	<25	22	20	28	26
	25-30	40	40	41	43
	30-35	25	27	18	23
	>35	13	13	13	8
Diabetes		36	40	23	34
Pre-op Afib		12	9	21	22
COPD		15	14	18	17
CVD		14	14	13	17
PVD		17	19	7	18
Frailty		4	3	6	8
EF<40		15	15	11	20
NYHA	I	35	44	13	13
	II	23	23	29	21
	III	26	20	42	42
	IV	15	13	17	24
HGB (g/L)	<115	50	52	49	38
	115-135	33	32	32	41
	>135	17	15	19	21
Pre-op creatinine	<115	74	76	78	62
(μmol/L)	115-140	14	13	12	18
	140-160	5	5	3	8
	>160	7	7	7	11
Redo sternotomy		7	4	19	11
Status	Elective	47	44	59	44
	In-house	41	41	36	43
	Urgent	9	11	4	10
	Emergent	3	4	2	3

*BMI* Body mass index, *HGB* Hemoglobin, *Afib* Atrial Fibrillation, *COPD* Chronic Obstructive Pulmonary Disease, *CVD* Cerebro Vascular Disease, *PVD* Peripheral Vascular Disease, *EF* Ejection Fraction, *NYHA* New York Heart Association.

**Table 3:** Frequency of MACE and MACE components in the model development cohort

**Variable**		**All Procedures**	**CABG**	**AVR/MVR/MVrpr**	**CABG + Valve**
		**n=4270**	**n=3095**	**n=696**	**n=479**
		**(%)**	**(%)**	**(%)**	**(%)**
MACE*		15.7	13.0	14.0	32.0
Mortality		4.2	3.3	3.3	11.5
Acute renal failure		6.5	5.5	5.5	14.0
Any stroke		2.9	2.3	2.4	7.3
	Transient (<24 h)	1.7	0.9	1.2	3.1
	Permanent	1.7	1.4	1.3	4.2
Infection		8.0	7.1	6.3	16.1
	Deep sternal Wound infection	1.1	1.0	0.1	2.9
	Sepsis	2.8	2.4	1.9	7.3
	Pneumonia	6.5	5.7	5.3	13.6

*MACE—Major Adverse Cardiac Event defined as in-hospital death, stroke (persisting at discharge or transient), acute renal failure (new post-operative renal failure or acute on chronic (>50% increase from baseline creatinine)), or infection (sepsis, pneumonia, or deep sternal wound infection).

### Model development

Assessment of the variance inflation for each variable revealed that no variable exceeded 4.0 allowing all variables to remain in the final model (see Additional file 1: Table 5).

By LOESS regression, the squared transformation of the continuous variable age had the most linear relationship with the logodds of the outcome (see Additonal file 1: Figure 2-3). Hemoglobin and creatinine had non-linear relationships with the outcome despite transformations. The inflection points of the natural variable were taken to create categorical variables. Hemoglobin inflection points were 115 and 135 and creatinine was 115, 140, and 160.

A total of 16 variables remain in the logistic regression model (Table 4). Significant predictors of MACE include variables such as Frailty, BMI >35, all levels of Creatinine, DM, Emergent and Urgent status and CABG + valve procedure type. Model beta coefficients are presented in the Additional file 1: Table 5.

**Table 4:** Logistic regression for MACE in a CABG/Valve population

**Variable**		**Odds ratio**	**95% confidence limit**
Age square		1.025	1.02-1.03
Female		0.8	0.6-1.0
PVD		1.6	1.2-1.9
Frailty		1.7	1.2-2.5
BMI	25-30	1.0	-
	<25	1.2	0.9-1.5
	30-35	1.2	0.9-1.5
	>35	1.5	1.1-2.1
NYHA I		1.0	-
NYHA II		1.2	0.9-1.6
NYHA III		1.2	0.9-1.5
NYHA IV		1.3	1.0-1.8
HGB <115		1.2	1.0-1.5
HGB 115-135		1.8	1.3-2.3
HGB >135		1.0	-
Creatinine	<115	1.0	-
	115-140	1.3	1.1-1.7
	140-160	1.6	1.1-2.2
	>160	1.6	1.2-2.2
Preop Afib		1.4	1.1-1.8
Diabetes		1.5	1.1-1.8
EF < 40		1.3	1.0-1.7
COPD		1.2	0.9-1.5
CVD		1.2	1.0-1.6
HTN		1.1	0.9-1.5
Preop RF		1.3	0.9-1.9
Procedure	CABG	1.0	-
	Valve	1.2	0.9-1.5
	CABG + Valve	2.3	1.8-3.0
Surgical priority elective		1.0	-
	In-house	1.1	0.9-1.5
	Urgent	1.8	1.8-3.6
	Emergent	4.3	2.9-7.4
Redo		1.4	0.9-1.9

### Model performance

The concordance statistic for the logistic regression was 0.764, which is equivalent to an ROC of 76.4% (95% CI; 75–79). The Hosmer-Lemeshow goodness of fit statistic was not significant (p=0.3133).

**Figure 1:**
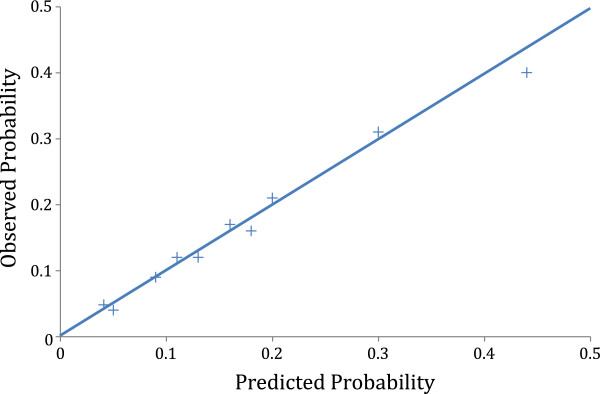
Calibration plot of observed risk versus predicted risk.

The deciles of observed over predicted probabilities of MACE are plotted (Figure 1). Each data points falls on or very near the ideal line indicating excellent calibration.

## Discussion

This paper outlines the development of a logistic regression model. Our model is unique for three reasons: 1) it performs well in a heterogeneous population including CABG, valve, and CABG + valve patients, 2) it predicts a composite outcome of quality indicators including death and major morbidities and, 3) it was developed for a contemporary cohort that represents a contemporary cardiac surgery practice. Also, we could not identify in the literature any models that include a CABG, valve and valve + CABG population with a composite end point.

Although many models exist that describe isolated CABG or isolated valve [1-9,16-18], their use is limited to only a percentage of the cardiac surgical population. EuroSCORE, perhaps the most widely recognized cardiac surgical predictive model, is a mixed population model that has had great success in research and as a quality improvement tool [11,18]. However, its derivation cohort is over 15 years old restricting its applicability to a contemporary practice. Our model is derived from a 2004–2009 cohort allowing for more current application. The EuroSCORE II has failed to perform better then the original model in certain procedure type subsets [13,14]. Furthermore both EuroSCORE models only predicts mortality, and not other important quality indicators.

Provided the model performs well, the advantage of a heterogeneous population model exceeds that of single-procedure models in its ability to describe to majority of surgical patients and can help facilitate quality improvement efforts. However, certain predictive variables, such as descriptions of coronary artery disease or valve disease severity, cannot be included in the model, as they do not apply to the entire model derivation cohort. This might be problematic, as some variables like left main disease have previously been identified in the literature as highly important variables to include in CABG mortality models [25]. Although our model cannot contain this variable (as it would be entirely co-linear with CABG patients), it does contain other clinically relevant variables previously used in other published models [1-9,16-18].

Modeling composite outcomes allows for a broader prediction of important post-operative events rather than being limited to a single outcome. Also, the components of our composite are each identified as quality indicators in cardiac surgery [15] allowing for improved clinical validity. The benefit of a correctly chosen composite outcome allows for more detailed description and prediction of the clinical population increasing the clinical relevance of the model.

The model discrimination is high with a ROC 76.4% (75–79, 95% CI) that exceeds published recommendations [26] and is similar to other published cardiac surgical models [1-9,16-18]. This indicates that the model has good predictive ability. The bootstrap procedure, a form of internal validation [27,28], allows for estimation of the 95% confidence interval. The tight 95% confidence interval provides a further estimation of reliability of the model. The calibration plot allows for a visual representation of the model’s performance (Figure 1). The observed to expected (O/E) data points fall on or very near the ideal line indicating excellent calibration of the model. The model performed well for each individual procedure subset as well (isolated CABG, isolated valve, CABG/valve).

## Conclusions

We provide a statistically and clinically relevant model that is an essential tool in the era of quality improvement. The model includes heterogeneous procedures and a composite outcomes increasing its utility.

As the profile of the cardiac surgery patients changes, so must the predictive models used to describe this group. This is of utmost importance in the field of quality assessment and improvement. Models such as the one reported in this manuscript, assists many QI techniques. They can be used to perform pre-operative predictive risk matching to allow for comparison of matched groups and can risk adjust surgeon specific surgical outcomes for report carding [24,26,29]. The benefit of the mixed population and composite end points facilitates describing a contemporary clinical practice so that QI efforts are more productive. Developers of such models must be dedicated to upholding high statistical standards, so that the QI efforts actually benefit the patients. As QI efforts become a staple in cardiac surgery practice, models such as these are essential in propelling advancement in this field and improving outcomes for our patients.

## Abbreviations

BMI: Body mass index; CABG: Coronary artery bypass grafting; CL: Confidence limits; QEII HSC: Queen Elizabeth II health science center; QI Quality: Improvement; LOESS: Locally weighted scatter plot smoothing; MACE: Major adverse cardiac events; NYHA: New York heart association; O/E: Observed/Expected; OR: Odds ratio; VIF: Variance inflation factor; WHO: World Health Organization.

## Competing interests

The authors declare that they have no competing interests.

## Authors’ contributions

CRH carried out the data analysis and authored the manuscript. KJB provided statistical support. CRH, KJB and RB participated in the design of the study. All authors contributed to the editing of the manuscript and have approved the final manuscript.

## Supplementary Material

Additional file 1 Statistical Methods. Click here for file
