# Supplementary material for: Development of a predictive model for major adverse cardiac events in a coronary artery bypass and valve population
Source: J Cardiothorac Surg. 2013 Jul 30;8:177. doi: 10.1186/1749-8090-8-177 (PMC3751077; doi:10.1186/1749-8090-8-177)
Supplement: Additional file 1 — Statistical Methods. [file 1749-8090-8-177-S1.docx]

**Appendix**

**Statistical Methods**

*Colinearity*

Multi-colinearity of the variables included in the model was assessed via the variance inflation factor (VIF). A VIF of greater than 4.0 indicates excessive multi-colinearity (1) and any variables exceeding this threshold would be reconsidered for analysis or collapsed.

*Linearity and Transformations*

As logistic regression assumes that every predictor variable X has a linear relationship with the logodds of the dependent variable Y (2, 3), scatterplots of the continuous variables, creatinine, age, and pre-operative hemoglobin, were examine to assess the relationship of the candidate variables with MACE. Locally weighted least squares regression (LOESS), which estimates regression surfaces by implementing nonparametric methods (4), was applied to each variable and transformation scatterplot to visually represent the linear relationship. A smoothing bandwidth of 0.1-0.5 was applied to each LOESS regression and the variable or transformation with the most linear relationship was presented to the model (5). Transformations including squared, square root, and spline were also plotted. If the variable still violated the linear assumption after transformations, the inflection points from the loess regression were used to create categorical variables. Inflection points indicate a change in the slope of the relationship between the variables and the outcome. Subsequently, by using the inflection points as cutoff values, each category has a homogeneous relationship with MACE.

The relationship of BMI with the dependent variable is traditionally parabolic.

*Model Validation*

As the bootstrap procedure provides nearly unbiased estimates of the models accuracy (2), it was used as a validation tool. The model was run on two hundred sub-samples of 63.2% of the model cohort with replacements. Confidence limits (95%) for the ROC were taken from the 2.5^th^ and 97.5^th^ percentiles of the bootstrap procedure (6).

References

36. OBrien RM. A caution regarding rules of thumb for variance inflation factors*. Quality & Quantity* 2007;41:673-90.

28. Harrell FE, Lee KL, Mark DB. Tutorial in Biostatistics Multivariable Prognostic Models: Issues in Developing Mdels, Evaluating Assumptions and Adequacy, and Measuring and Reducing Errors*. Stats Med* 1996;15:361-87.

37. LaValley MP. Logistic regression*. Circulation* 2008;117:2395

24. Cleveland WS. Robust locally weighted regression and smoothing scatter- plots*. J Amer Stats Ass* 1979;74:829-36.

38. Cohen RA. An introduction to PROC LOESS for local regression*. SAS Institute Inc. Proceedings of the Twenty-Fourth Annual SASR Users Group International Conference* 1999.

18. Nowicki ER, Birkmeyer NJ, Weintraub RW, et al. Multivariable prediction of in-hospital mortality associated with aortic and mitral valve surgery in northern new england*. Ann Thorac Surg* 2004;77:1966-77.

| **Variable** | **Definition** |
| --- | --- |
| Age | Patient age at the time of surgery |
| Gender | Male or Female |
| Body Mass Index | Calculated in kilograms and centimeters. |
| Diabetes | Any history of Diabetes Mellitus, regardless of duration |
| Pre-op Afib | Any previously documented history of Atrial Fibrillation |
| COPD | Any preivous documented history of Chronic Obstructive Pulmonary Disease |
| CVD | Any Transient Ischemic Attack, Cerebrovascular Accident/Stroke, history of cerebrovascular surgery, or any carotid disease. |
| PVD | Whether the patient has Peripheral Vascular Disease, as indicated by claudication; amputation for arterial insufficiency; aorto-iliac occlusive disease reconstruction; peripheral vascular bypass surgery, angioplasty, or stent; documented AAA. |
| Frailty | Any deficiency in the Katz index of Activities of Daily Living (independence in feeding, bathing, dressing, transferring, toileting, and urinary continence), as well as independence in ambulation (no walking aid or assist required) or any clear evidence of a previous diagnosis of dementia by a physician. |
| EF<40 | Ejection fraction measured less than 40% by any modality. |
| NYHA (I-IV) | New York Heart Association Class. I = Patients with cardiac disease but without limitation of physical activity. II = Patients with cardiac disease resulting in slight limitation of physical activity (fatigue, palpitations, dyspnea, or anginal pain). III = Patients with cardiac disease resulting in marked limitation of physical activity. IV = Patients with cardiac disease resulting in inability to carry on any physical activity without discomfort. Symptoms of cardiac insufficiency or of the anginal syndrome may be present even at rest. |
| Hemoglobin | Most recent hemoglobin level prior to day of surgery. |
| Pre-op Creatinine | Highest preop serum creatinine for this admission. |
| Redo Sternotomy | Any history of previous surgery that traversed the anterior mediastinum. |
| Surigical Priority | Elective [stable at home], in-house [requiring hospitalization until the time of surgery], urgent [requiring surgery within 24 hours to minimize further clinical deterioration], or emergent [no delay in surgery]. |
| Procedure | Any Coronary artery bypass grafting, aortic valve replacement or repair with/without CABG, or Mitral valve replacement or repair with/without CABG. |

**TABLE #4-Model Variable Definitions**

**Figure #2- Loess Regression for Age and Age Transformations**

Loess regression was performed for the variable age un-transformed (A), with a squared transformation (B), and a spline transformation (C). A smoothing bandwidth of 0.3 was applied to each regression. The squared transformation visually yielded the most linear relationship with the outcome.

| A | 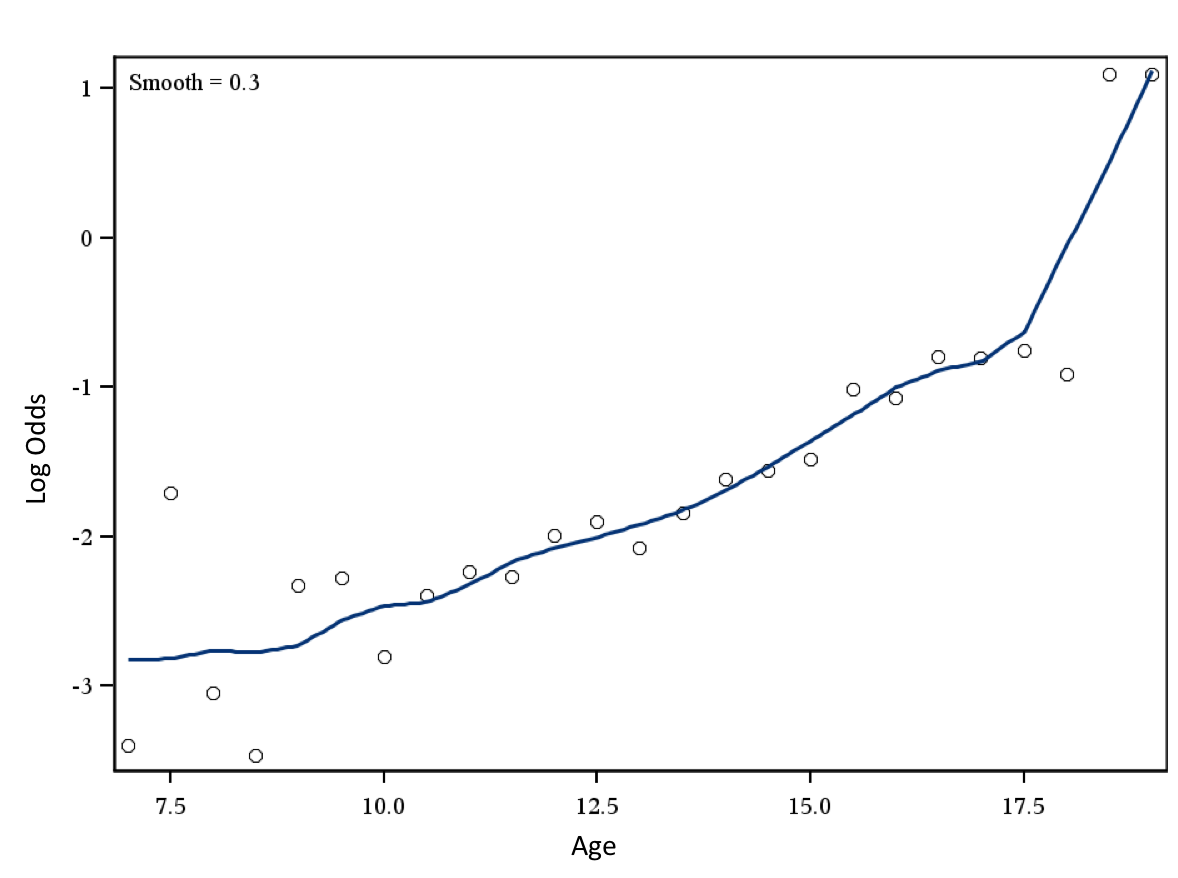 |
| --- | --- |
| B | 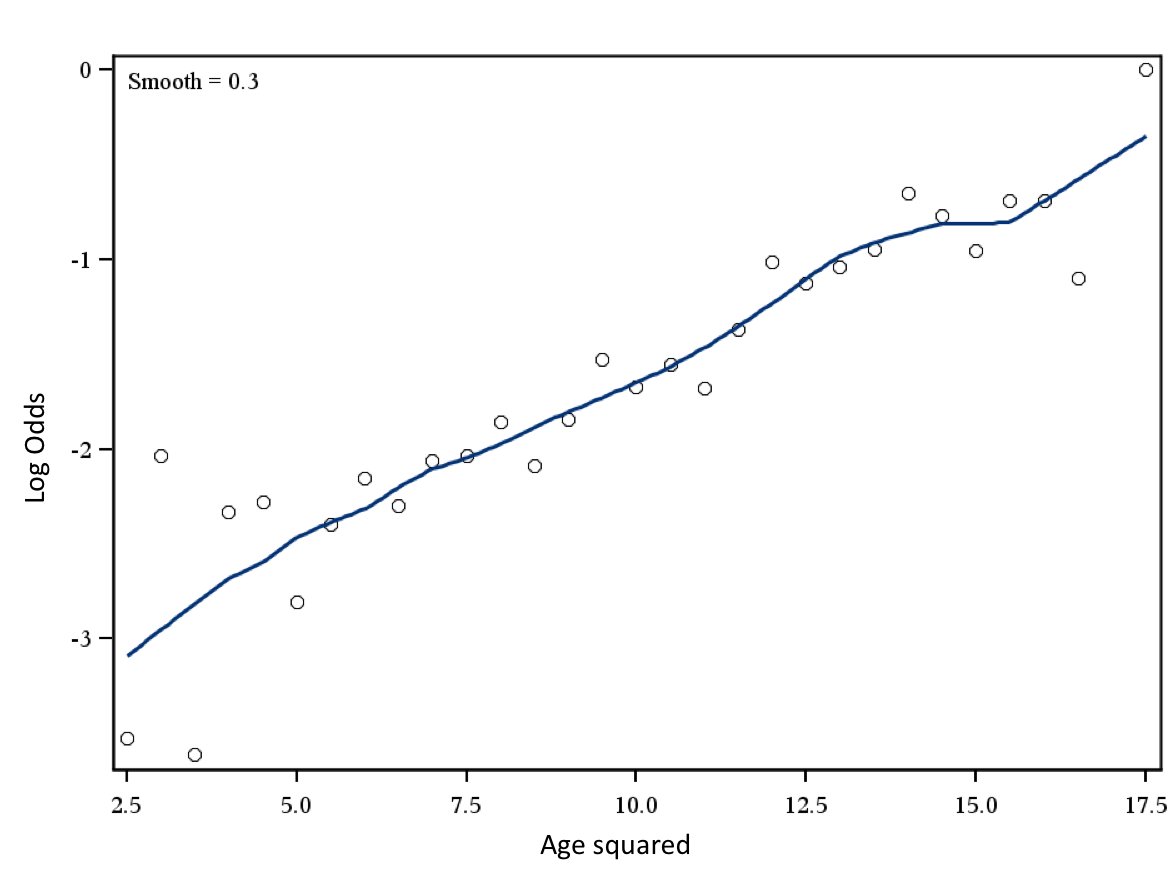 |
| C | 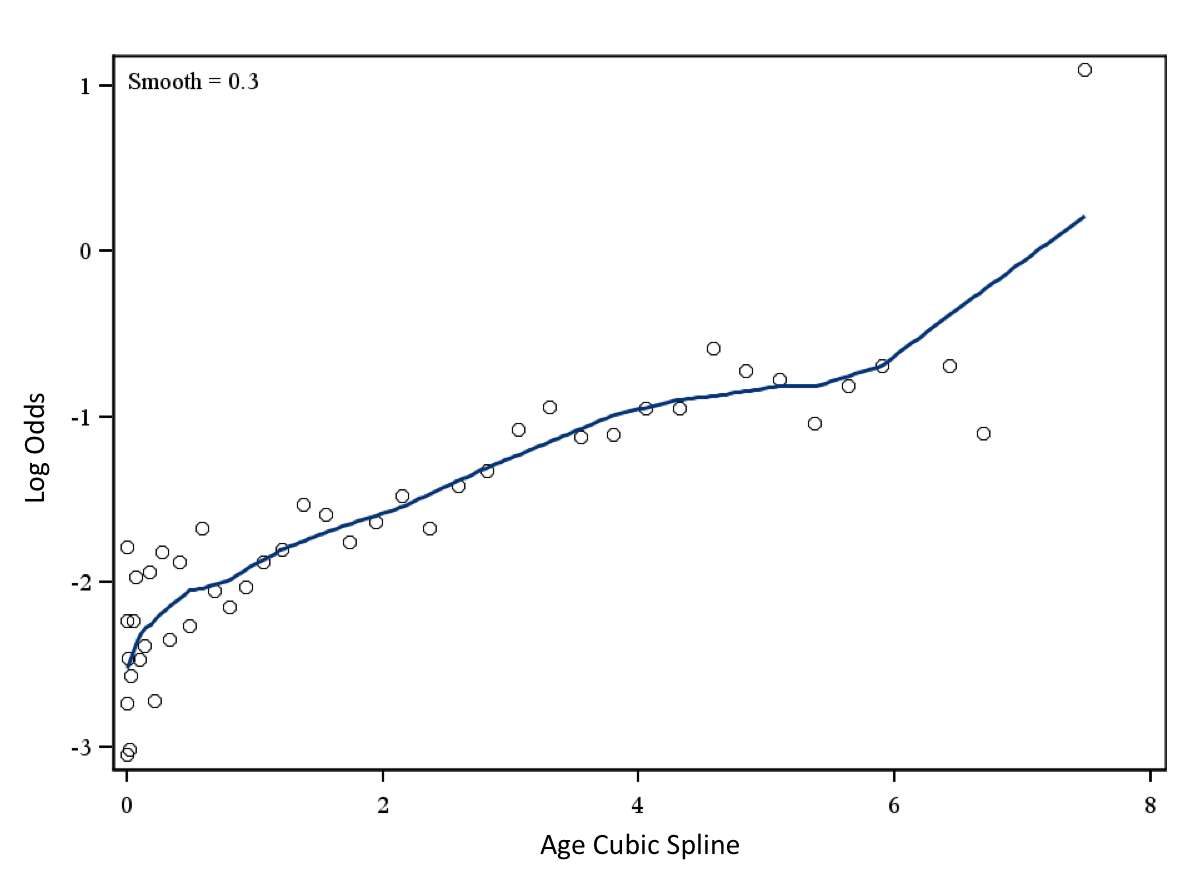 |

**Figure #3—Loess Regression for the Continuous Variable Pre-operative Hemoglobin**

(A) Loess regression was applied to the variable hemoglobin. (B) As no transformations yielded a suitable linear relationship, categories were created based on inflection points in the slope of the regression line. The red line highlight the inflection points. A smoothing bandwidth of 0.4 was applied to the regressions.

| A | 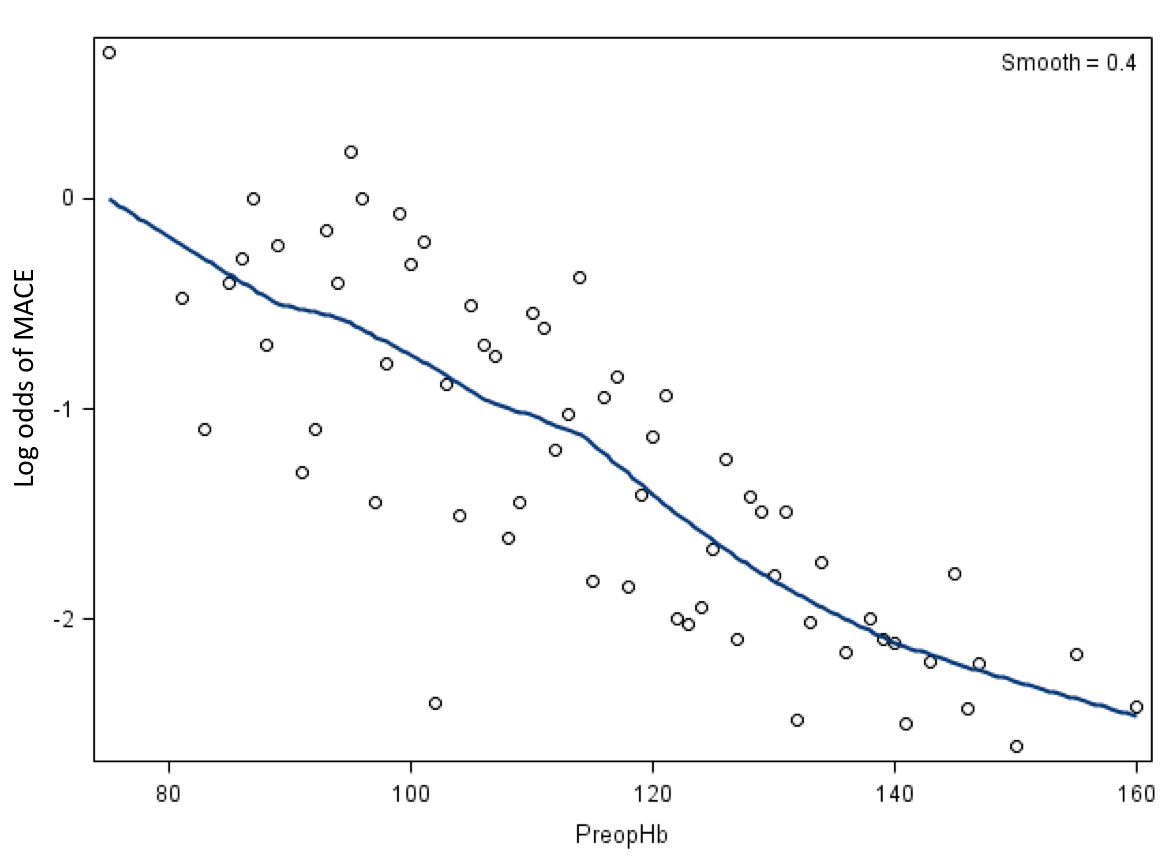 |
| --- | --- |
| B | 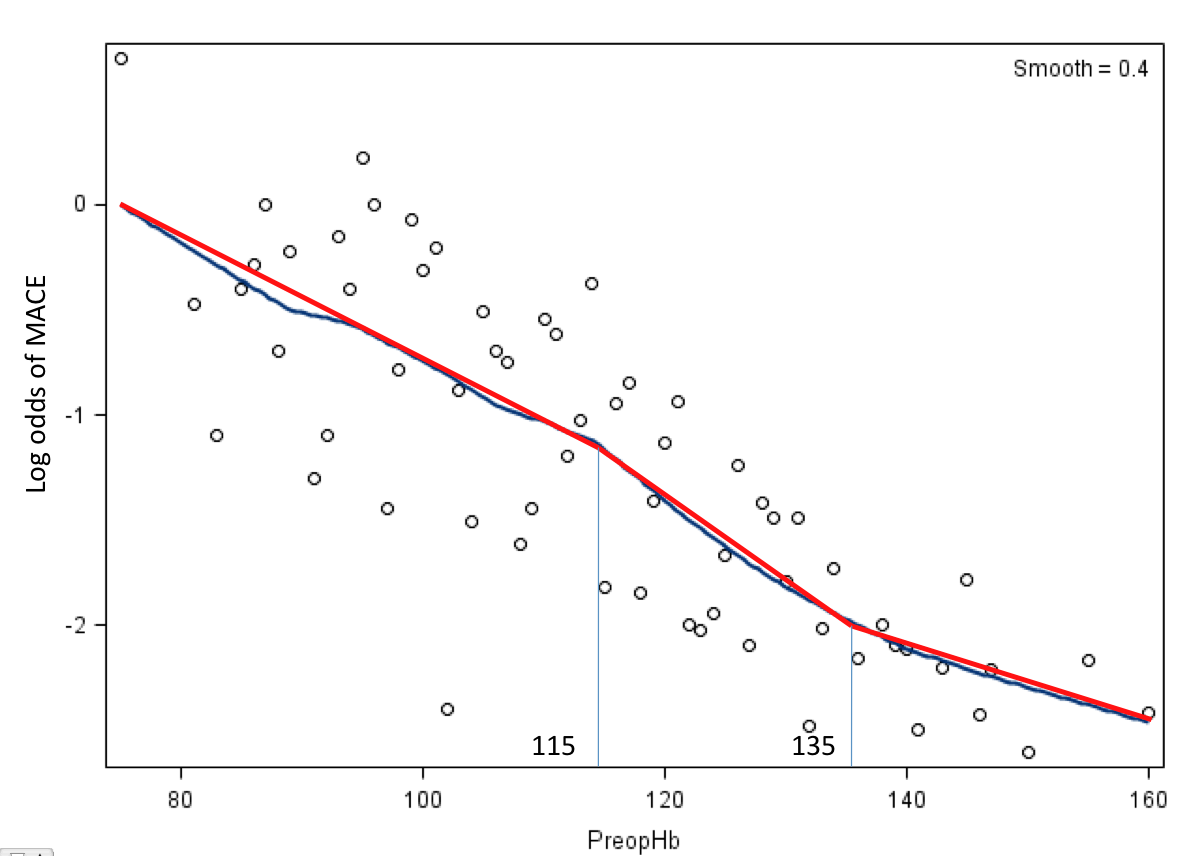 |

| **Variable** | **Coefficients**  **(95% CI)*** |
| --- | --- |
| Intercept | -4.28 (-4.71, -3.88) |
| Age Square | 0.025 (0.01, 0.03) |
| Female | -0.22 (-0.46, -0.011) |
| PVD | 0.43 (0.22, 0.64) |
| Frailty | 0.42 (0.18, 0.83) |
| BMI 25-30 | - |
| <25 | 0.18 (-0.06, 0.45) |
| 30-35 | 0.17 (-0.05, 0.42) |
| >35 | 0.39 (0.05, 0.71) |
| NYHA I | - |
| NYHA II | 0.19 (-0.08, 0.49) |
| NYHA III | 0.12 (-0.08, 0.35) |
| NYHA IV | 0.28 (0.02, 0.62) |
| HGB <115 | 0.20 (-0.01, 0.40) |
| HGB 115-135 | 0.56 (0.28, 0.82) |
| HGB >135 | - |
| Creatinine <115 | - |
| 115-140 | 0.29 (0.02, 0.49) |
| 140-160 | 0.43 (0.08, 0.78) |
| >160 | 0.47 (0.12, 0.78) |
| Afib | 0.36 (0.09, 0.58) |
| Diabetes | 0.39 (0.27, 0.57) |
| EF < 40 | 0.28 (0.06, 0.49) |
| COPD | 0.13 (-0.14, 0.37) |
| CVD | 0.19 (-0.03, 0.44) |
| Procedure CABG | - |
| Valve | 0.13 (-0.13, 0.41) |
| CABG + Valve | 0.83 (0.58, 1.08) |
| Status Elective | - |
| In-House | 0.21 (0.003, 0.44) |
| Urgent | 0.86 (0.58, 1.13) |
| Emergent | 1.44 (0.98, 1.85) |
| Redo Sternotomy | 0.27 (-0.01, 0.57) |

* 95% CI taken from the 2.5^th^ and 97.5^th^ percentile from the bootstrap procedure

**Table #4—Beta Coefficients for the MACE Model**
